# Improving nutrition outcomes through enhanced allocative efficiency of investments in 24 high risk counties in Kenya: An Optima Nutrition modelling study

**DOI:** 10.1371/journal.pone.0323391

**Published:** 2025-05-27

**Authors:** Morris Ogero, Nick Scott, Veronica Kirogo, Anthony K. Ngugi

**Affiliations:** 1 Department of Infectious Disease Epidemiology, London School of Hygiene and Tropical Medicine, London, England; 2 Department of Population Health, Aga Khan University, Nairobi, Kenya; 3 Modelling and Biostatistics Group, Burnet Institute, Melbourne, Australia; 4 Division of Nutrition & Dietetics, Ministry of Health, Kenya; Nelson Mandela University, SOUTH AFRICA

## Abstract

**Introduction:**

Undernutrition remains a significant global challenge, severely impacting children’s development and growth. To address this, the Sustainable Development Goals target a substantial reduction in stunting and wasting by 2025. Achieving these goals requires scaling up evidence-based nutritional interventions; however, limited budgets pose challenges in funding all necessary programs. To assist policymakers in making informed decisions, the World Bank developed the Optima Nutrition Modelling tool, which optimizes the allocation of nutrition investments. Kenya, with its high prevalence of stunting, was the focus of this study. Using the Optima Nutrition model, we aimed to (1) assess the impact of scaling up evidence-based nutrition interventions and (2) determine how existing resources could be optimized to reduce stunting, wasting, and anemia in children under five and anemia in pregnant women across 24 counties with the poorest nutrition outcomes.

**Methods:**

Utilizing the Optima Nutrition model, we analyzed demographic and intervention data to assess the impact and allocation of interventions. Scenario analyses and optimization techniques were employed to reallocate resources and evaluate their potential impact on reducing undernutrition.

**Results:**

When scaled up to 95% coverage and maintained until 2030, across the counties the interventions resulted in median relative reductions of 14.6% in stunting prevalence, 23% in wasting prevalence, 20.6% in anaemia prevalence among children and 64.2% in anaemia prevalence among pregnant women. For stunting, the optimized scenarios prioritized infant and young child feeding education, vitamin A supplementation, lipid-based nutrition supplements for children, and balanced energy-protein supplementation and multiple micronutrient supplementation for pregnant women. For wasting, cash transfers was prioritized. For anaemia in children, long-lasting insecticide treated bednets and IFA fortification of maize were prioritized. For anaemia in pregnant women, multiple micronutrient supplementation, iron and folic acid supplementation and long-lasting insecticide-treated bed nets were prioritized.

**Conclusion:**

This study provided a comprehensive assessment of the prevalence of stunting, wasting, and anemia among children under five years in 24 counties in Kenya. The Optima model suggested that scaling up nutrition-specific interventions under the same baseline budgets could lead to significant reductions in stunting, wasting, and anemia in Kenya. Additionally, the study identified interventions that should be prioritized during nutrition intervention resource allocation.

## Introduction

The scourge of undernutrition is a global challenge that has a potential of impairing child brain development, linear growth, cognitive health and other child growth milestones [1]. It is estimated that almost half of under-five annual mortality is attributable to various forms of malnutrition [2]. It is on this basis that the Sustainable Development Goals (SDGs) aim to reduce the global prevalence of stunting to <15% and wasting to <5% by 2025 [3]. However, in UNICEF’s 2019 report [4], one third of children under 5 are either stunted, wasted or overweight, and the remaining two thirds are at risk of malnutrition because of the poor quality diets. It is therefore likely that in most countries, the SDG target on malnutrition may not be achieved unless there is an accelerated scale-up of evidence-based nutritional interventions. Research has quantified the resources required to scale up these interventions and the feasibility of reaching these global targets on time [5–7].

A variety of interventions have been proven to be effective in addressing undernutrition, but limited budgets mean that it is not possible to fund them all. Therefore, difficult decisions need to be made about how to prioritize interventions to achieve the best health outcomes with the available funding. To help policy makers and program implementers address these questions, World Bank developed the Optima Nutrition tool [8,9] which is an open-source mathematical model that estimates the optimal allocation of nutrition investments across interventions to address stunting, wasting in under-five children, and anemia in women of reproductive age.

In Kenya, 26% of children under the age of five are stunted, according to the 2014 Kenya Demographic and Health Survey (KDHS). Alarmingly, 24 of the country’s 47 counties report stunting prevalence rates exceeding 26%. These higher rates are strongly associated with factors such as low maternal education levels, poverty as reflected in lower household wealth quintiles, and suboptimal breastfeeding practices[10]. Wasting, characterized by low weight for height, affects 12.5% of Kenyan children under five, while anemia impacts nearly 50% of this age group. These nutritional challenges are primarily driven by inadequate access to nutritious food, poor sanitation and hygiene practices, and limited availability of healthcare services. Without targeted, effective interventions, the prevalence of wasting and anemia is unlikely to improve.

## Methods

### Optima model

Optima is a suite of open-source decision-support tools developed by the World Bank and Optima Consortium for Decision Science. Optima models are used to inform public health policy decisions by simulating the potential impact of different interventions on disease transmission, prevalence, and mortality. Optima models have been used to inform policy decisions for a variety of diseases, including HIV, tuberculosis, malaria, hepatitis C, and nutrition [8,9].

In this study we used the Optima Nutrition modelling tool to estimate how a fixed budget could be optimally allocated across various evidence-based nutrition interventions to minimize poor nutrition outcomes (stunting, wasting, and anemia). The Optima model shares a similar intervention impact framework to Lives Saved Tool (LiST) [11] to estimate the impact of scaling up interventions. The model also includes a mathematical optimization algorithm that can be used to calibrate budgets and intervention coverage to achieve optimal budget allocations for different objectives.

In each county, the model tracks the number of women of reproductive age (15–49 years) who can become pregnant and give birth. After birth, children are tracked until five years of age across age bands: < 1 month, 1–5 months, 6–11 months, 12–23 months and 24–59 months. Children in each age-band are categorised by height-for-age (stunting) status, weight-for-height (wasting) status, anaemia status, breastfeeding practice, and household’s economic status -relative to the poverty line. Children can exit the model at age 60 months or via death. For infants younger than one month, death can be caused by diarrhoea, pneumonia, meningitis, asphyxia, sepsis, prematurity, and other factors. Mortality risks for these causes depend on the child’s breastfeeding, height-for-age, weight-for-height, and anaemia status. Mortality among pregnant women can be due to antepartum haemorrhage, intrapartum haemorrhage, postpartum haemorrhage, hypertensive disorders, sepsis, abortion, embolism, and other direct or indirect causes. The risk of such deaths is based on the woman’s anaemia status.

When intervention coverages are increased or decreased in the model, this leads to changes in projected outcomes (stunting, wasting or anemia) based on the intervention effectiveness parameters (effect sizes). The evidence for intervention impacts against nutritional outcomes are obtained from systematics reviews and meta-analyses of efficacy and effectiveness studies that also inform WHO guidelines on management of nutrition-related conditions [12,13].

The epidemiological model is overlaid with an economic framework, with the spending on each intervention linked to the intervention’s coverage via a cost function. A mathematical optimization algorithm is then used to incrementally shift funding between interventions to determine the allocations that achieve the best health outcomes with least cost. The Optima model has been used previously by the World Bank and others to support resource allocation decisions across multiple settings [14].

### Data assembly

The Optima Nutrition model requires data on demographics, epidemiology and intervention coverage and costs of delivering such interventions to community. Demographic data includes age-specific population sizes and projected births, which was obtained from Kenya National Data Archive [15]. Epidemiological data which includes nutritional indicators such as prevalence of stunting/wasting/anaemia, breastfeeding practices, mortality rates, birth outcomes and food consumption patterns were obtained from Kenya Demographic and Health Survey (KDHS) of 2014 [16], Standardized Monitoring and Assessment of Relief and Transitions (SMART) nutrition survey data [17], Kenya Health Information System [18], Multiple Cluster Indicators Survey [19], Comprehensive poverty report from Kenya National Bureau of Statistics [20], Global Burden of Disease study [21], World Bank Investment Framework for Nutrition [22], Population and Housing Census (2019) and relevant research papers. Where country-specific estimates for a given nutritional indicator epidemiological data were not available, national averages were obtained from Scott et al (2020)[9].

Evidence-based nutrition-specific interventions included in the model are shown in Table 1 for stunting, Table 2 for wasting, and Table 3 for anaemia. Costing studies were not available for most interventions, so their unit costs were estimated using an ingredients-based approach. The unit cost of each intervention included commodity costs (at market prices), health provider’s time and nutrition’s supply chain costs (estimated from UNICEF’s supply catalogue [23]). In total we assembled data for 66 baseline coverage indicators across different child age categories and women of reproductive age, 30 nutritional interventions and their unit costs for each of the 24 counties.

**Table 1:** Coverage of nutrition-specific interventions target with impact on stunting.

Intervention	Target population	Effects	Median baseline coverage among 24 counties	Median unit costs of delivering an intervention
Balanced energy protein supplementation	Pregnant women below the poverty line	Reduces risk of SGA birth outcomes	6.85% (IQR: 5.70% -7.57%)	USD 2.8(IQR: 2.08–3.6)
Multiple micronutrient supplementation in pregnancy	Pregnant women	Reduces anaemia in pregnant women Reduces risk of SGA birth outcomes	3.8% (IQR: 2.27%-5.23%)	USD 0.1(IQR: 0.1–0.03)
Public provision of complementary foods	Children 6–23 months below the poverty line	Reduces the odds of stunting	3.5% (IQR: 2.97% - 4.60%)	USD 0.1(IQR: 0.1–0.1)
Prophylactic zinc supplementation	Children 1–59 months	Reduces diarrhea incidence Reduces diarrhea and pneumonia mortality	86.55%(IQR:78.67%- 96.73%)	USD 0.1(IQR: 0.1–0.1)
Vitamin A supplementation	Children 6–59 months	Reduces diarrhoea incidence and mortality	12.85%(IQR:12.1% - 13.97%)	USD 0.1(IQR: 0.1–0.1)
Infant and young child feeding education (IYCF)	Children <23 months	Increases exclusive breastfeeding (children <6 months) increases partial breastfeeding (children 6–23 months), improves complementary feeding practices to reduce stunting and wasting.	15.2%(IQR:10.2% - 19.33%)	USD: 0.5(IQR: 0.18–0.77)
Cash Transfer			2.05%(IQR: 1.12–2.95)%	USD: 43.5(IQR: 29.45–61.7)
IFA fortification of maize			50%(IQR: 50%-50%)	
IFA fortification of rice			42.2%(IQR: 38.98% - 43.62%)	
IPTp			15.1%(IQR: 7.5% - 28.47%)	USD 0.3(IQR:0.3–0.3)
Lipid-based nutrition supplements coverage			4.9%(IQR 4.6% - 5.15%)	USD 1.6(IQR: 1.17–2.05)
Micronutrient powder			1.15%(0.58% - 2.5%)	USD 0.1(IQR: 0.1–0.03)
Zinc + ORS			12.85%(IQR: 12.07% - 15%)	USD 0.5(IQR: 0.5–0.6)

**Table 2:** Nutrition-specific interventions targeting with impact on wasting.

Intervention	Target population	Effects	Median baseline coverage among 24 counties
Cash transfers	All children below the poverty line	Reduces the incidence of SAM Reduces the incidence of MAM Indirectly reduces SAM mortality. Indirectly reduces MAM mortality	2.05% (IQR: 1.12% -2.95%)
IFA fortification of maize			50% (IQR: 50–50)
IFA fortification of rice			42.2% (IQR: 38.98–43.62)
Treatment of severe acute malnutrition (SAM)	Children experiencing SAM	Reduces the prevalence of SAM; Reduces mortality	1.7% (1.28% - 2.22%)
Vitamin A supplementation			12.85% (IQR: 12.07–15)
Zinc + ORS			12.85%(IQR: 12.07% - 15%)
Zinc supplementation coverage.			86.55% (IQR: 77.12–97.12 0

**Table 3:** Nutrition-specific interventions targeting with impact on anemia.

Intervention	Target population	Effects	Median Coverage among 24 counties
IFA supplementation for pregnant women	Pregnant women.	Reduces anaemia	4% (IQR: 2.6% - 5.23%)
IFA supplementation	Non-pregnant Women of Reproductive Age	Reduces anaemia	22.8% (IQR: 21.97% - 23.5%)
Multiple micronutrient supplementation	Pregnant women	Reduces anaemia. Reduces risk of SGA birth outcomes	3.8% (2.27%-5.23%)
Intermittent Preventive Treatment of Malaria for Pregnant Women (IPTp)	Pregnant women in areas where there is malaria risk	Reduces anaemia. Reduces SGA birth outcomes	15.1% (IQR: 9.6% - 20.0%)
Food fortification	Everyone	Reduces anaemia Reduces neonatal mortality	50% (IQR: 50% -50%)
Long-lasting insecticide-treated bed nets	Everyone in areas where there is malaria risk	Reduces anaemia. Reduces SGA birth outcomes	39.5% (IQR: 27.4% - 45.77%)

### Validation of the assembled data, and training on the usage of the Optima model

The data compiled underwent a thorough review and validation process during workshops involving teams from county health departments and the department of nutrition and dietetics within the national Ministry of Health. Any potential discrepancies in the data were identified and resolved by cross-checking with the original data sources. Moreover, the Optima modelling team conducted comprehensive presentations of their study findings during trainings and data validation exercises with the Division of Nutrition and Dietetics (DND) at the Ministry of Health. This initiative was then extended to 24 counties, organized into four groups of six. Each county had five key health professionals, including the County Nutrition Coordinator, M&E/planning personnel, County Health Records Information expert, along with professionals in Child Health, and reproductive/maternal health, who received training on the application of the Optima model and provided additional validation of the data specific to their counties. In total, ninety-six officers from both national and county levels, representing the 24 counties, participated in these workshops.

### Data analysis

**Fig 1:**
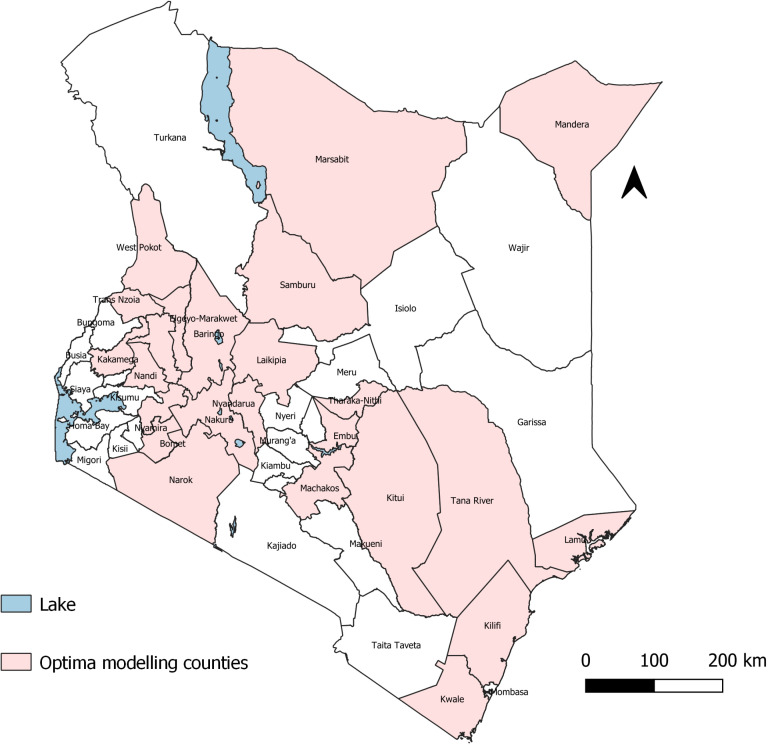
Locations of the counties included in this study.

The data analysis for this study utilized the Optima Nutrition model to evaluate the allocative efficiency of nutrition investments across 24 high-burden counties in Kenya as shown in Fig 1. County-specific data-books, created as macro-enabled Microsoft Excel spreadsheets, were developed and uploaded onto the Optima platform for each county. These data-books included baseline data on stunting, wasting, and anemia prevalence, intervention coverage, and costs.

We conducted two types of analyses:

1. Scenario Analyses

Scenario analyses were performed to estimate the potential impact of scaling up all interventions to 95% coverage over a five-year period (2021–2025), with the heightened coverage sustained until 2030. The outcomes examined included stunting, wasting, and anemia in children under five and anemia among pregnant women. A comparative analysis was conducted between the impacts of this scale-up scenario and a business-as-usual(baseline) scenario for each county.

1. Optimization Analyses

Optimization analyses were carried out to determine the most effective allocation of the estimated total current spending to achieve various objectives. These objectives included minimizing the prevalence of stunting, wasting, and anemia in children under five, as well as anemia in pregnant women. The optimization considered only those interventions with evidence-based direct or indirect effects on the outcomes of interest, as identified from the literature. The analysis was projected across a 10-year period (2021–2030), starting from a 2020 baseline. The optimized outcomes were compared against a business-as-usual scenario where spending patterns remained unchanged throughout the modelling period.

For both analyses, the Optima Nutrition model calculated the cost-effectiveness of interventions, simulated potential health gains, and provided estimates for reallocated budgets and projected outcomes under each scenario. These outputs included optimized intervention coverage levels, reallocated resources, and reductions in stunting, wasting, anemia, and child mortality.

## Interventions considered for each nutritional outcome

### Modelling Stunting

The Optima model categorizes children based on their height-for-age measures and a criterion from a reference population. Within these categories, the two lowest ones, severe and moderate, are identified as stunting, aligning with the standards set by the World Health Organization (WHO). Stunting is influenced by several risk factors including sub-optimal birth outcomes (e.g., pre-term or small for gestational age births), previous instances of stunting, inadequate feeding practices, and occurrences of diarrhoea. Notably, stunting increases the risk of mortality in children suffering from ailments like diarrhoea, pneumonia, and measles.

To mitigate the risks associated with stunting, the model considers various interventions each with a direct or indirect impact on stunting. For instance, scaling up vitamin A supplementation could lead to a reduction in diarrhoea incidence, indirectly curbing stunting, and the likelihood of mortality among children under five. Similarly, an increased implementation of multiple micronutrient supplementation among pregnant women could decrease adverse birth outcomes like small-for-gestational-age, which are risk factors for stunting and neonatal mortality. The relationship between stunting risk factors, nutritional interventions, and their correlation with mortality is visually depicted in supplementary file Fig 1.

### Modeling wasting

The model classified children into four distinct categories based on their weight-for-height distribution, aligning with the WHO reference population: severe acute malnutrition (SAM), moderate acute malnutrition (MAM), mild acute malnutrition, and normal. Children falling into either SAM or MAM categories are categorized as “wasted”. It’s worth noting that within each age group, the status of wasting in one period doesn’t influence the prevalence of wasting in subsequent periods since wasting is an acute condition. Supplementary file Fig 2 illustrates those reductions in diarrhoea incidence and improvements in birth outcomes, such as term/pre-term births and appropriate for gestational age (AGA) or small for gestational age (SGA), contribute to reducing the prevalence of wasting.

**Fig 2:**
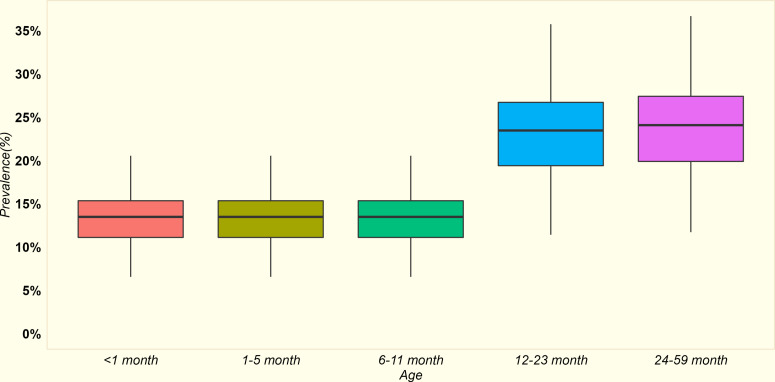
Baseline distribution of stunting (defined as moderate or severe stunting) across different age groups among the 24 counties.

Various interventions recognized for their impact on reducing wasting prevalence were taken into consideration. These interventions included the treatment of SAM, public provisions of complementary foods, lipid-based nutrition supplements, and social protection programs like cash transfers.

### Modelling anemia

The model considers the prevalence of anaemia within different sub-populations, specifically among children, pregnant women, and women of reproductive age. Anaemia during pregnancy has been associated with adverse birth outcomes and an increased risk of maternal mortality, primarily due to the higher probability of haemorrhage.

Supplementary Fig 3 illustrates various interventions considered within the model to address anaemia. These interventions include practices such as delayed cord-clamping of newborns, which proves beneficial in preventing anaemia by allowing infants to receive a larger volume of iron-rich blood from the placenta. Additionally, the model also incorporates the fortification of staple foods like maize, wheat, and rice with iron and folic acid. However, the coverage of this fortification within the community is limited to the fraction of the population that consumes these foods as their primary staple, a factor determined from consumption data.

## Results

### Stunting in children

As stunting is a chronic condition, its prevalence tends to escalate with age. Specifically, stunting rates for moderate to severe forms were noted at 12.9% for children under one year of age (interquartile range (IQR) across counties: 10.6% to 14.7%) and increased to 26.7% (IQR: 22.1% to 30.4%) for children aged 12–59 months, as illustrated in Fig 2.

We considered the following interventions in the stunting scenarios: long-lasting insecticide-treated bednets, lipid-based nutrition supplements, multiple micronutrient supplementation, balanced energy-protein supplementation, IPTp, zinc supplementation, vitamin A supplementation, IYCF, public provision of complementary foods, cash transfers, zinc for treatment + ORS, IFA fortification of maize, and IFA fortification of rice. The baseline coverage of these interventions varied considerably across the 24 counties as shown in Table 1. For example, balanced energy-protein supplementation for pregnant women had a median coverage of 6.85% with interquartile range across counties of 5.70% to 7.57%, while prophylactic zinc supplementation had a median of 86.55% coverage (IQR 78.67%- 96.73%) among the 24 counties. When these interventions were scaled up from their initial coverage rates to achieve 95% coverage and maintained until 2030, the model results suggested a significant increase in the number of alive and non-stunted children turning age 5 between 2020–2030, with an average increase of about 15%, ranging from 10.6% to 16.6% across the 24 counties.

**Fig 3:**
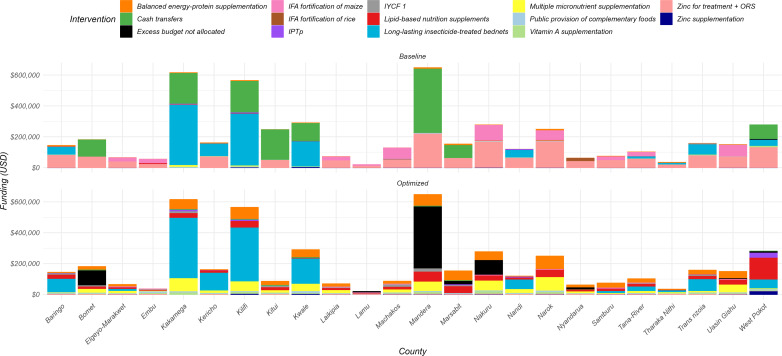
Allocative efficiency to minimize stunting prevalence across 24 counties. The baseline bars represent estimated spending on nutritional interventions aimed at reducing prevalence of stunting. Optimized bars depict the results of budget reallocation, leading to a reduction in stunting prevalence. This reallocation shifted resources, predominantly allocated to Zinc for treatment and ORS at baseline, to more effective interventions, including balanced energy-protein supplementation, multiple micronutrient supplementation, lipid-based nutrition supplements, infant and young child feeding education (IYCF) and vitamin A supplementation. Note that the size of bars does not necessarily reflect coverage due to different unit costs (e.g., vitamin A is prioritized but does not require much budget as it is cheaper than the other interventions). In instances where funding was sufficient to reach full coverage of all interventions that impact stunting, the remainder was classed as “Excess Budget not allocated.” These funds could be used to address the other nutritional outcomes.

In the optimization scenario, the optimization algorithm identified ways that stunting prevalence could be reduced with the current spending. In analyzing the nutritional interventions aimed at combating stunting, we found that the combined total baseline spending per county was estimated to be a median of USD 1202 (IQR: 538–3435) per annum, with primary allocations directed towards Zinc for treatment + ORS which was consistent across the counties. In an optimized scenario, the model reallocated and shifted resources to a more cost-effective mix of interventions, including balanced energy-protein supplementation, multiple micronutrient supplementation for pregnant women, infant and young child feeding education, vitamin A supplementation, and lipid-based nutrition supplements for children. The changes in resource allocation were substantial. For balanced energy-protein supplementation, coverage increased from 1.2% (IQR 0.8%-2.3%) at baseline to 19% (IQR 10.7%-29.5%) in the optimized scenario. Multiple micronutrient supplementation saw an increase from 0.8% (IQR 0.53%-1.1%) coverage at baseline to 21.1% (IQR 10.6%-26.9%). For lipid-based nutrition, coverage increased from 0.66% (IQR 0.46%-0.95%) at baseline to 14.3% (IQR 9.4%-18.9%) in the optimized scenario. See Fig 3 for more details. Coverage and costs of different interventions including their unit costs in the baseline and optimization scenarios are shown in supplementary file 1.

### Wasting in children

**Fig 4:**
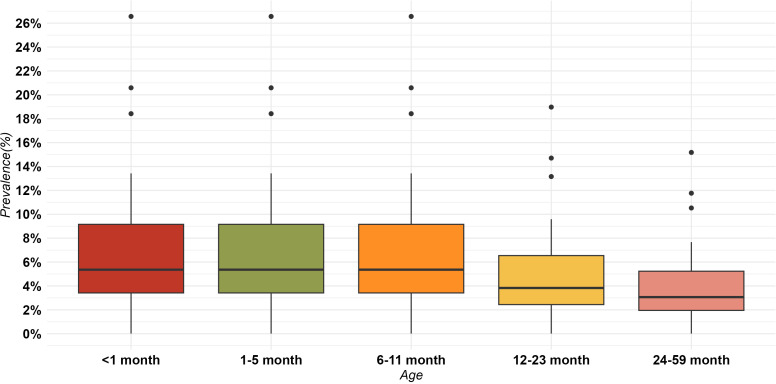
Distribution of wasting across different age groups among the 24 counties.

Across the 24 counties included in the study, the median rate of wasting among children under five was 4.86%, with an interquartile range of 2.98%-8.75%. More specifically, for children under one year, the rate of wasting was 5.36% (interquartile range 3.41%-9.16%) and 3.47% among 12–59-month-olds (interquartile range 2.3%-6.3%), as depicted in Fig 4.

We considered the following interventions in the wasting scenarios: cash transfer program targeting households below the poverty line, IFA fortification of maize/rice, long-lasting insecticide treated bed nets, treatment of severe acute malnutrition (SAM), vitamin A supplementation, Zinc for treatment + ORS and Zinc supplementation. Across the counties, these interventions showed varying median baseline coverages: 2.05% (IQR: 1.12%-2.95%) for cash transfers, 1.7% (IQR: 1.28% - 2.22%) for treatment of SAM, and 12.85% (IQR: 12.07–15.0%) for vitamin A supplementation among others as outlined in Table 2. When these interventions were scaled up from their initial coverage rates to achieve 95% coverage and maintained until year 2030, the model results suggested a noteworthy relative reduction in wasting prevalence of about 23% ranging from 20.8% to 29.1% within the 24 counties.

**Fig 5:**
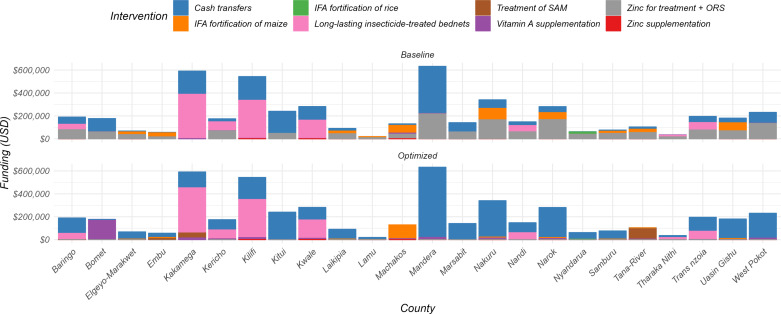
Allocative efficiency of resources to minimize wasting prevalence in 24 counties. Baseline bars denote the status quo allocations to nutritional interventions targeting wasting, and the optimized bars denote optimization results, where a reduction in wasting was achieved by increasing budget reallocation primarily to cash transfers in most counties.

For the wasting nutritional outcome, resources were predominantly assigned to cash transfers and zinc for treatment + ORS at baseline. However, these resources were significantly reallocated in the optimization scenario to minimize wasting prevalence. At baseline, cash transfers had a median allocation of 22% (IQR 10.8% - 37.2%) across counties, which increased to 92.02% (IQR 47.3% - 93.6%) in the optimized scenario, and vitamin A was scaled-up to full coverage in most countries. Conversely, the allocation for zinc + ORS shifted from 45.1% (IQR 39.3% - 52.8%) at baseline to a median of 0% across the 24 counties in the optimized scenario as shown in Fig 5.

### Anemia

**Fig 6:**
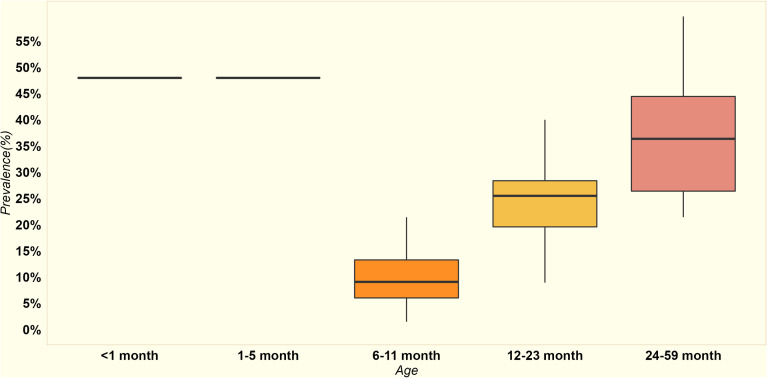
Distribution of under-five anemia prevalence across different age groups among the 24 counties.

The prevalence of anemia was notably high among different age groups. For children under five months old, the anemia prevalence stood at 47.9%. In the 6–11-month age group, the prevalence of anemia had a median value of 9%, (IQR: 6% to 13%). For children aged 12–23 months, the median anemia prevalence is higher at 25% (IQR: 20% to 28%). In the 24–59-month age group, the median anemia prevalence further increases to 36% (IQR: 26% to 44%) as shown in Fig 6. Among pregnant women, the prevalence of anemia was reported at 38.2%, with no variation observed across different age groups. This indicates a consistent and relatively high prevalence of anemia among pregnant women regardless of age.

We considered the following interventions in the anaemia scenarios: cash transfer, supplementation with iron and folic acid (IFA) in rice, fortification of food(rice/wheat/maize), Intermittent Preventive Treatment of Malaria for Pregnant Women (IPTp), iron and iodine fortification, long-lasting insecticide-treated bed nets, and multiple micronutrient supplementation for pregnant women. These interventions had varied baseline coverages. For instance, supplementation with IFA for pregnant women exhibited a median coverage of 4% across counties, varying from 2.6% to 5.23%. Additionally, IPTp started at a baseline coverage of 15.1% (IQR: 9.6%-20%). Upon scaling up interventions from their initial coverage rates to achieve 95% coverage and maintaining this level until 2030, the model outputs suggested a substantial 20.6% relative reduction in anemia prevalence among children. This reduction varied across the 24 counties, ranging from 9.5% to 30.3%. Similarly, anemia prevalence among pregnant women showed a noteworthy average relative reduction of 64.2%, ranging from 62.3% to 68.23%.

**Fig 7:**
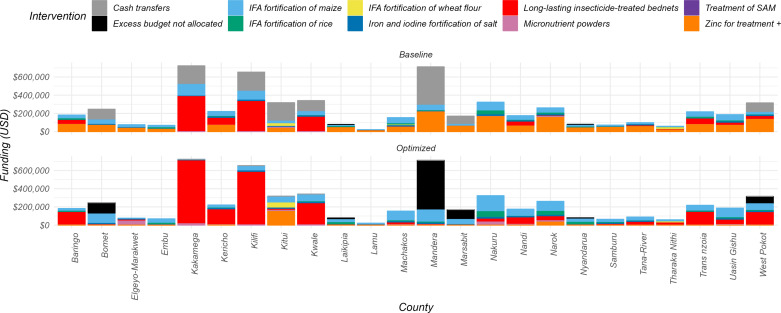
Allocative efficiency of resources towards the reduction of anemia prevalence in children in 24 counties. Baseline bars denote the status quo allocations to nutritional interventions, and the optimized bars denote optimization results, where a reduction in number of anemic children was achieved in the model by reallocating budgets primarily to LLINs and IFA fortification of maize. In the instances where funding was sufficient to reach full coverage of all interventions that impact anaemia in children, the remainder was classed as “Excess Budget not allocated.” These funds could be used to address the other nutrition outcomes.

In the optimization to reduce anaemia in children, the resources shifted to two main interventions: IFA fortification of maize and long-lasting insecticide-treated bed nets. At baseline, IFA fortification of maize had a median of 24.7% (IQR 17.5% - 29.4%) of the resources, which increased to 40.2% (IQR 22.1% - 54.6%) in the optimization scenario across counties. Resources for long-lasting insecticide-treated bed nets also changed from a median of 8.7% (IQR 2.5% - 30.2%) at baseline to 6.5% (IQR 6.4% - 59.5%) in the optimized scenario as shown in Fig 7. Notably, the model reallocated a substantial portion of resources away from the cash transfer program, particularly in the counties of Bomet, Mandera, Marsabit, and West Pokot, to fund these interventions.

**Fig 8:**
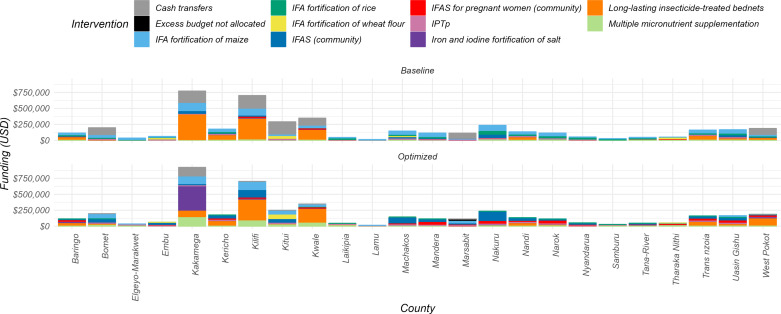
Allocative efficiency of resources towards reduction of anemia prevalence in pregnant women in 24 counties. Baseline bars denote the status quo allocations to nutritional interventions, and the optimized bars denote optimization of the available resources to effective interventions.

In the optimization to reduce anemia in pregnant women, the reallocation strategy shifted budgets to four key interventions. Multiple micronutrient supplementation saw an increase from a baseline median of 0.94% (IQR 0.63% - 1.4%) to an optimized 2.6% (IQR 1.5% - 13%) across counties. Iron and iodine fortification of salt rose from a baseline median of 2.3% (IQR 1.4% - 3.2%) to 2.6% (IQR 1.8% - 3.7%) in the optimized scenario. The allocation for IFAS (Iron and Folic Acid Supplements) for pregnant women increased significantly from 0.68% (IQR 0.48% - 0.90%) to 15.5% (IQR 1.4% - 22.4%). Lastly, the resources for long-lasting insecticide-treated bed nets were adjusted from 13.7% (IQR 3.4% - 41.4%) at baseline to 22% (IQR 7.4% - 45.9%) in the optimization as shown in Fig 8.

## Discussion

Overall, this study estimated the prevalence of stunting, wasting, and anemia among children under five years in 24 counties in Kenya. We also investigated the impact of scaling up various evidence-based nutrition-specific interventions for stunting, wasting, and anemia, as well as optimizing the current estimated spending on these interventions. It is noteworthy that county nutrition budgets are often allocated non-uniformly across interventions without necessarily being informed by evidence, leading to inefficiencies in resource use. Scaling up nutrition-specific interventions to 95% coverage led to notable median reductions in malnutrition outcomes: a 14.6% reduction in stunting, a 23% reduction in wasting, a 20.6% reduction in anemia among children, and a 64.2% reduction in anemia among pregnant women. These results reaffirm the effectiveness of scaling interventions such as lipid-based nutrition supplements, multiple micronutrient supplementation for pregnant women, and vitamin A supplementation, which address critical nutrient deficiencies contributing to malnutrition. The variation in reductions across counties reflects differences in baseline coverage rates, intervention effectiveness, and contextual factors such as socioeconomic conditions and healthcare access.

The optimization algorithm within the Optima Nutrition model also identified ways for reducing stunting, wasting, and anemia through programmatic shifts with the same spending. For each of the focus outcomes (stunting, wasting & anemia), these analyses have identified the most cost-effective mix of evidence-based nutrition interventions which yield improved nutrition outcomes under the baseline funding scenarios, and should therefore be prioritized in the county decision-making. For stunting, resources were reallocated from less impactful interventions, such as zinc supplementation for diarrhea management, to balanced energy-protein supplementation, multiple micronutrient supplementation for pregnant women, and lipid-based nutrition supplements for children. This reallocation aligns with evidence suggesting that direct nutritional interventions addressing maternal and child nutrient deficiencies are more effective at reducing stunting [24,25]. The optimized scenario also emphasized the importance of infant and young child feeding education (IYCF), which promotes appropriate feeding practices to mitigate the risk of chronic undernutrition.

The optimization of resources for minimizing wasting prevalence prioritized cash transfers and IFA fortification of maize, which can influence birth outcomes (which are a risk factor for wasting). Cash transfers offers a form of social protection that enables households to meet their basic nutritional needs and other essential requirements. Both interventions can be integrated into a comprehensive strategy to tackle wasting effectively. This approach underscores the importance of leveraging both direct nutritional interventions and broader socioeconomic support to improve public health outcomes.

It was surprising to observe that treating severe acute malnutrition was not found to be cost-effective against wasting, considering it is implemented alongside other interventions such as lipid-based nutrition supplements, cash transfer and public provision of complementary feeding. This is because treatment of SAM is defined as treating children until they reach a weight-for-height of three standard deviations below the WHO Child Growth Standards median, at which point their mortality risks drastically reduce, yet they are still considered wasted if they meet the standards of the SDG target. Therefore, even after they have received successful treatment, they may remain in the category of ‘wasted’ in terms of the SDG target. This finding (SAM treatment not being cost-effective) is consistent with previous similar multi-country study [9]. The other major point is that the model illustrates that wasting prevention interventions can be more cost-effective than treatment, which is significantly more expensive.

We also evaluated the cost-effectiveness of various nutritional interventions for addressing anaemia in children and pregnant women. The model’s optimization algorithm allocated a significant portion of the budget towards long-lasting insecticide-treated bed nets (LLITNs) for children, reflecting their high effectiveness in reducing anemia, particularly in malaria-endemic regions. LLITNs effectively combat anemia by mitigating malaria transmission, a major cause of childhood anemia. Moreover, LLITNs offer additional health benefits by reducing the risk of mosquito-borne diseases like dengue fever and lymphatic filariasis. In addition, among pregnant women, the model prioritized iron and iodine fortification, IFAS, LLITNs, and multiple micronutrient supplements due to their low unit costs and substantial impact on anemia reduction. These interventions effectively address iron deficiency, a primary cause of anemia in pregnant women.

### Implications of the study findings

This study’s demonstration of the substantial reductions in stunting, wasting, and anemia achievable through scaling up nutrition-specific interventions reinforces the importance of prioritizing these interventions in national and county-level nutrition strategies. By investing in interventions with proven effectiveness for reducing stunting, such as vitamin A supplementation, infant and young child feeding education, lipid-based nutrition supplements for children, balanced energy-protein supplementation and multiple micronutrient supplementation for pregnant women, interventions with proven effectiveness for prevention of wasting such as cash transfers, or LLITNs for anemia in children, governments can make meaningful progress towards reducing the prevalence of these conditions and improving child health outcomes. The utilization of the Optima Nutrition model to identify the most cost-effective interventions for each nutritional condition provides a valuable tool for guiding resource allocation decisions. By prioritizing interventions with the highest impact per dollar spent, policymakers can ensure that limited resources are used most efficiently to maximize the reduction of malnutrition. Future research should focus on developing and evaluating cost-effective nutrition-sensitive interventions and strategies for integrating these interventions into existing health systems. The study team identified a need to engage with policy makers and nutrition program planners at National and county levels to consider how incremental progress toward the optimal budget allocations can be achieved in practice. Additionally, there is need to explore ways to implement and test on-the-ground impact of these model recommendations on poor nutritional outcomes in the study counties. By expanding the evidence base on nutrition-sensitive approaches, researchers can contribute to the development of more comprehensive and effective nutrition policies and programs.

### Limitations

Evaluating the individual unit costs of many nutrition interventions that are embedded in health systems and provided alongside other health services can be difficult. These costs include commodities, supply chain, health provider time, infrastructure/equipment, health information systems, and expenditures for supplementary health programmes. Additionally, this study did not take into consideration “nutrition-sensitive” interventions which aim to address social, environmental, and health-related nutrition determinants. Prior studies have also shown that merely relying on nutrition-specific interventions will not suffice to reach the SDG targets, further underlining the necessity to invest in evidenced-based nutrition-sensitive programs [2,6]. The lack of data on coverage of such interventions at county level further complicates the assessment of baselines and progress made in implementation.

## Conclusion

This study assessed the prevalence of stunting, wasting, and anemia among children under five in 24 high-risk counties in Kenya and evaluated the impact of optimizing budget allocations for nutrition-specific interventions. Scaling up targeted interventions like balanced energy-protein supplementation, multiple micronutrient supplementation, and cash transfers can significantly reduce these conditions. The study also underscores the value of using optimization models, such as Optima Nutrition, to improve resource efficiency. Future efforts should focus on integrating nutrition-sensitive interventions and testing the real-world application of these strategies within existing health systems

## Supporting information

Supplementary files Fig 1 to Fig 3 The files show the schematic views of the relationship between nutritional interventions, risk factors, and mortality. **Adopted from the Optima Nutrition modelling user guide(https://optimamodel.com/docs/Optima%20Nutrition%20User%20Guide%20Feb2019.pdf**)
(DOCX)

## Data sharing statement

Data assembled and modelled by study are available upon a reasonable request to the institutional data governance committee via email nbi.krhdss-dph@aku.edu.
