# Correction: Improving nutrition outcomes through enhanced allocative efficiency of investments in 24 high risk counties in Kenya: An Optima Nutrition modelling study

**DOI:** 10.1371/journal.pone.0331557

**Published:** 2025-09-03

**Authors:** Morris Ogero, Nick Scott, Veronica Kirogo, Anthony K. Ngugi

There are errors in the Funding statement. The correct Funding statement is as follows: This study was funded by the Bill & Melinda Gates Foundation, Grant Number GCA/MNCH/Round6/031/001.
