# Supplementary material for: Improving nutrition outcomes through enhanced allocative efficiency of investments in 24 high risk counties in Kenya: An Optima Nutrition modelling study
Source: PLoS One. 2025 May 27;20(5):e0323391. doi: 10.1371/journal.pone.0323391 (PMC12112240; doi:10.1371/journal.pone.0323391)
Supplement: Supplementary files Fig 1 to Fig 3 — Adopted from the Optima Nutrition modelling user guide(https://optimamodel.com/docs/Optima%20Nutrition%20User%20Guide%20Feb2019.pdf) (DOCX) [file pone.0323391.s001.docx]

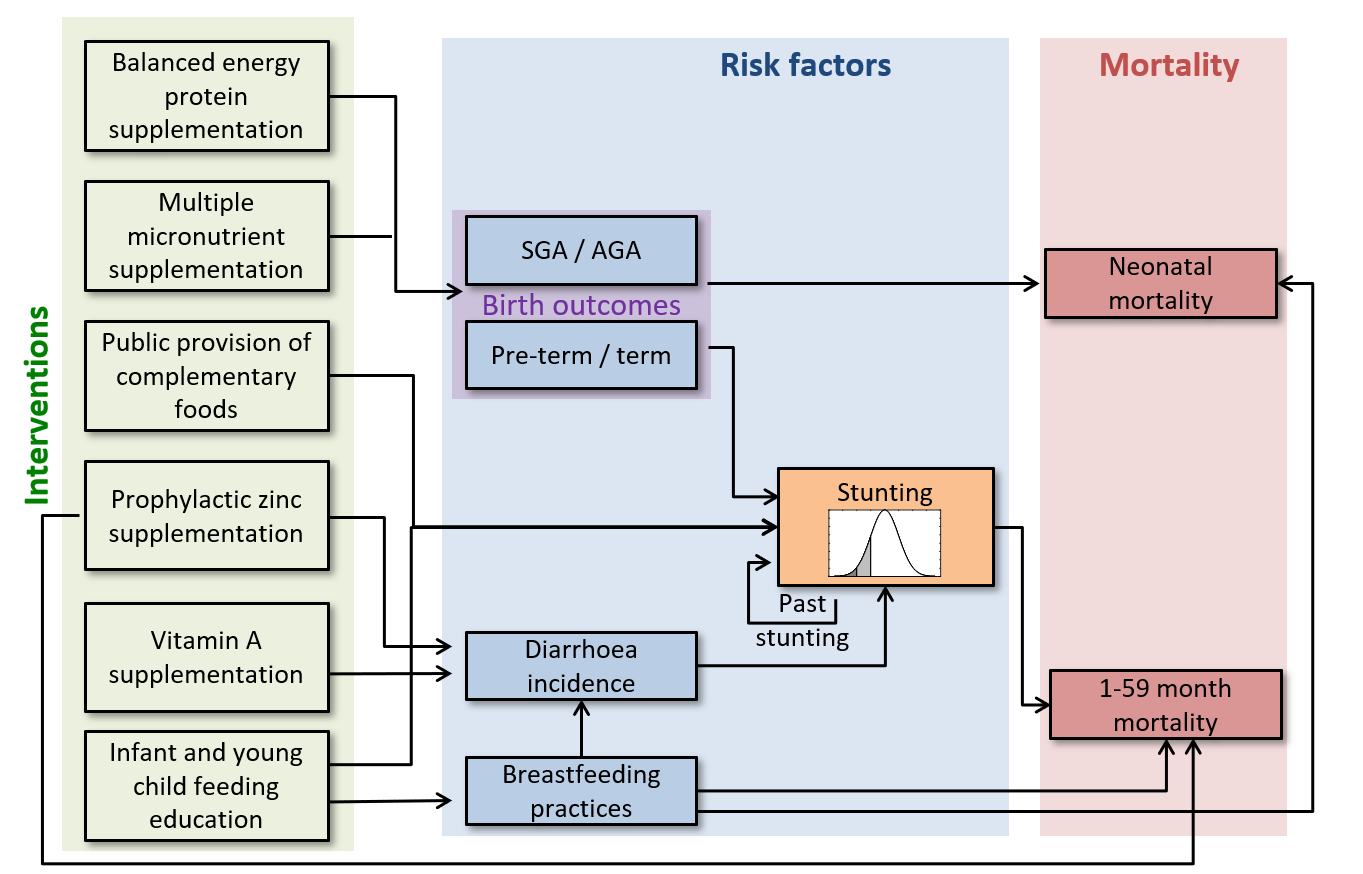


**Supplementary Figure 1: Schematic view of the relationship between stunting nutritional interventions, risk factors, and mortality. Adopted from the Optima Nutrition modelling user guide(**[**https://optimamodel.com/docs/Optima%20Nutrition%20User%20Guide%20Feb2019.pdf**](https://optimamodel.com/docs/Optima%20Nutrition%20User%20Guide%20Feb2019.pdf) **)**

***
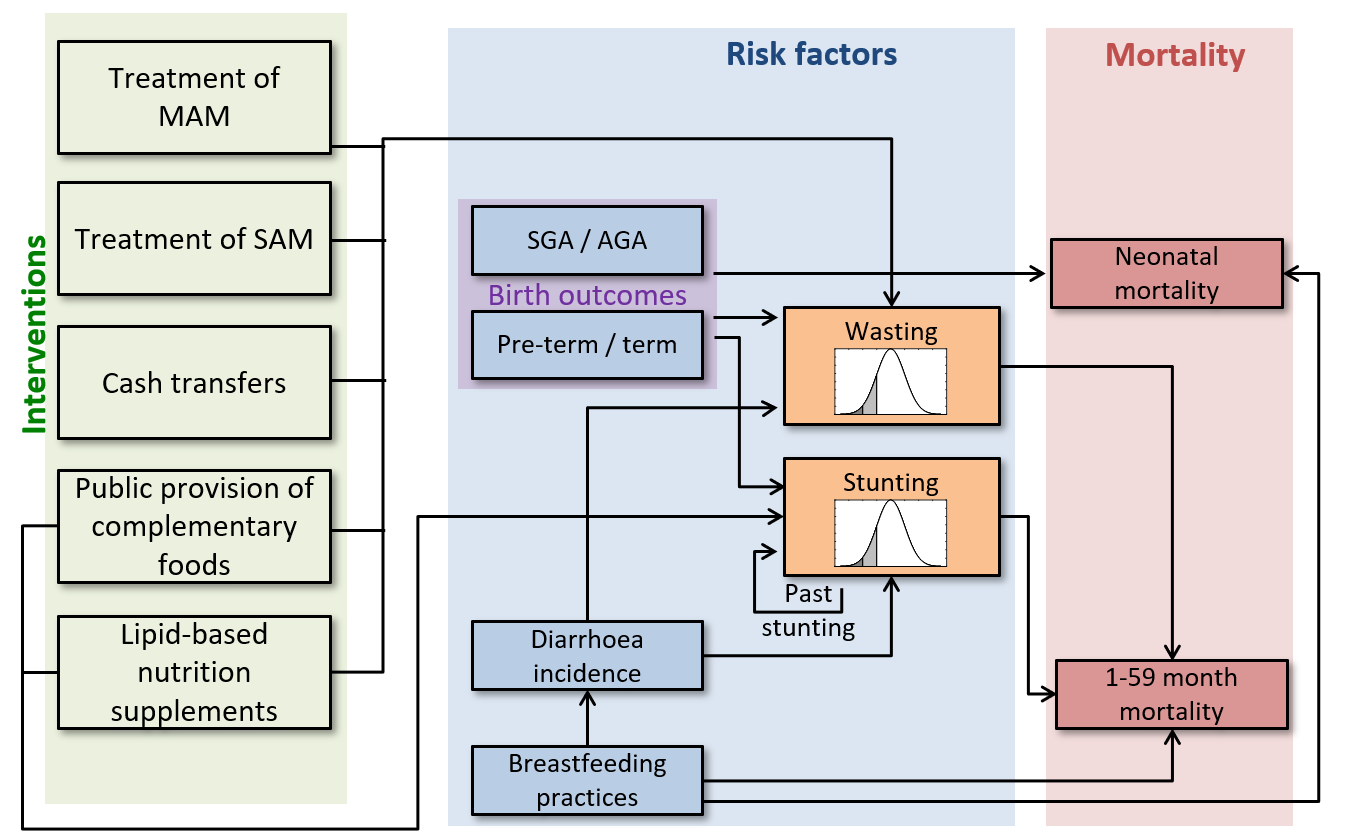
***

**Supplementary Figure 2: Schematic view of the relationship between nutritional interventions for wasting, risk factors, and mortality. Adopted from the Optima Nutrition modelling user guide(**[**https://optimamodel.com/docs/Optima%20Nutrition%20User%20Guide%20Feb2019.pdf**](https://optimamodel.com/docs/Optima%20Nutrition%20User%20Guide%20Feb2019.pdf) **)**

#
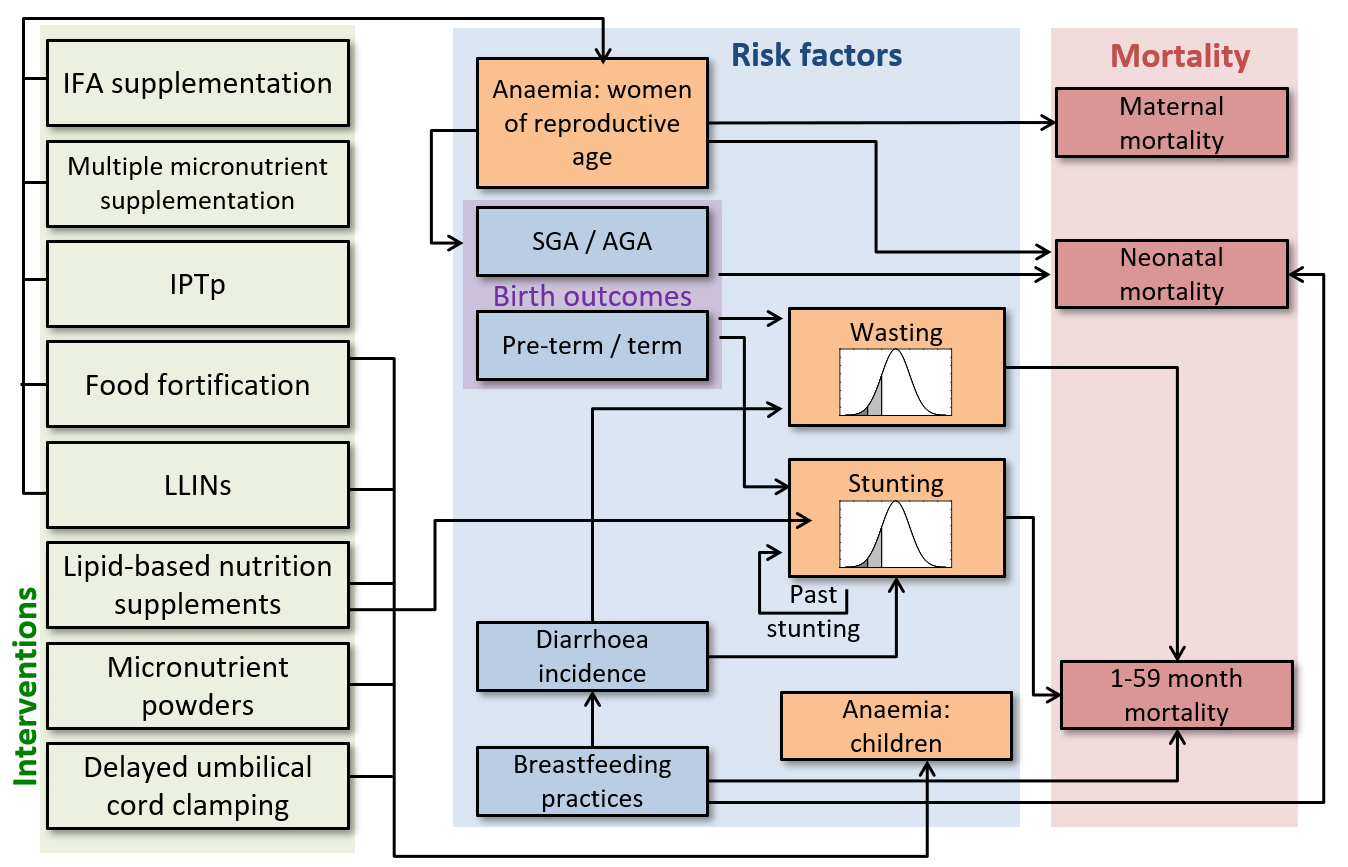
 Supplementary Figure 3: Relationship between nutritional interventions for anemia, risk factors and mortality: Key: Intermittent Preventive Treatment of Malaria for Pregnant Women (IPTp), Long-lasting insecticide-treated bednets(LLINs), Iron Folic Acid(IFA). Adopted from the Optima Nutrition modelling user guide(<https://optimamodel.com/docs/Optima%20Nutrition%20User%20Guide%20Feb2019.pdf> )
